# Frailty status changes are associated with healthcare utilization and subsequent mortality in the elderly population

**DOI:** 10.1186/s12889-021-10688-x

**Published:** 2021-04-01

**Authors:** Chia-Ming Li, Chih-Hsueh Lin, Chia-Ing Li, Chiu-Shong Liu, Wen-Yuan Lin, Tsai-Chung Li, Cheng-Chieh Lin

**Affiliations:** 1 Department of Family Medicine, National Taiwan University Hospital, Bei-Hu Branch, Taipei, Taiwan; 2 School of Medicine, College of Medicine, China Medical University, No. 100, Sec. 1, Jingmao Rd., Beitun Dist, Taichung City, 406040 Taiwan ROC; 3 Department of Family Medicine, China Medical University Hospital, Taichung, Taiwan; 4 Department of Medical Research, China Medical University Hospital, Taichung, Taiwan; 5 Department of Public Health, College of Public Health, China Medical University, Taichung, Taiwan; 6 Department of Healthcare Administration, College of Medical and Health Science, Asia University, Taichung, Taiwan

**Keywords:** Aged, Frailty, Health care, Utilization

## Abstract

**Background:**

This study determined (1) whether a change in frailty status after a 1 year follow up is associated with healthcare utilization and evaluated (2) whether a change in frailty status after a 1 year follow up and health care utilization are associated with all-cause mortality in a sample of Taiwan population.

**Methods:**

This work is a population-based prospective cohort study involving residents aged ≥65 years in 2009. A total of 548 elderly patients who received follow-ups in the subsequent year were included in the current data analysis. Fried frailty phenotype was measured at baseline and 1 year. Information on the outpatient visits of each specialty doctor, emergency care utilization, and hospital admission during the 2 month period before the second interview was collected through standardized questionnaires administered by an interviewer. Deaths were verified by indexing to the national database of deaths.

**Results:**

At the subsequent 1 year follow-up, 73 (13.3%), 356 (64.9%), and 119 (21.7%) elderly participants exhibited deterioration, no change in status, and improvement in frailty states, respectively. Multivariate logistic analysis showed the high risk of any type of outpatient use (odds ratios [OR] 1.94, 95% confidence interval [CI] 1.02–3.71) among older adults with worse frailty status compared with those who were robust at baseline and had unchanged frailty status after 1 year. After multivariate adjustment, participants with high outpatient clinic utilization had significantly higher mortality than those with low outpatient clinic visits among unchanged pre-frail or frail (hazard ratios [HR] 2.79, 95% CI: 1.46–5.33) and frail to pre-frail/robust group (HR 9.32, 95% CI: 3.82–22.73) if the unchanged robustness and low outpatient clinic visits group was used as the reference group.

**Conclusions:**

The conditions associated with frailty status, either after 1 year or at baseline, significantly affected the outpatient visits and may have increased medical expenditures. Combined change in frailty status and number of outpatient visits is related to increased mortality.

**Supplementary Information:**

The online version contains supplementary material available at 10.1186/s12889-021-10688-x.

## Background

An aging population is considered one of the most important demographic phenomena worldwide and is frequently referred to as a determinant of healthcare utilization [1, 2]. Community-dwelling older adults face the high risk of becoming frail. In particular, 13.6% of non-frail community older adults become frail after 3 years of follow-up [3]. Frail older adults are vulnerable to adverse health problems, including falls, delirium, fractures, disabilities, hospitalization, institutionalization, and mortality [4–9]. Several tools used to define frailty include the frailty index [10], Fried’s frailty phenotype (FFP; Cardiovascular Health Study) [4], the FRAIL scale [11], the Study of Osteoporotic Fractures Index [5], Edmonton Frailty Scale [12], and the Tilburg Frailty Indicator [13]. All older adults may be screened for frailty during clinical decision-making to provide more patient centered care, prevent iatrogenic harm, and deliver preventive care [14]. Many studies have demonstrated the effectiveness of preventing or reducing frailty levels in community-dwelling older adults [15, 16]. However, a meta-analysis showed that interventions for frail community-dwelling older adults have no significant effect on adverse outcomes [17]. Frailty is an important risk factor for the health of older adults, thus, epidemiology, natural course, intervention, challenges to healthcare policies concerning frailty, and the effects of frailty on older adults should be investigated.

Previous studies have shown that frail patients are more likely to visit outpatient clinics or consult doctors [18–22], visit the emergency room [18, 22–24], be admitted to hospitals [18, 20, 22, 24–28], and use community services [18, 19]. Frailty signifies high healthcare costs [24, 29–31] and long-term care costs [32]. However, frailty status is a dynamic process that may change after a certain period [33, 34]. Only a few studies have investigated the association between frailty status changes and medical utilization. Sirven et al. found that an elevated frailty index was associated with an increase in specialist practitioner visits [35]. We aimed to offer additional evidence and explore the relationship between medical utilization and frailty change.

Frailty has been proven to be associated with mortality [4, 6, 7, 9, 10]. The rate of change in frailty [22] and frailty transition [36–39] have been associated with mortality in some studies. Liu et al. found that worsening frailty and remaining frail increased painful death risk after 3 years of follow-up among 11,165 Chinese older adults [37]. One study involving 1171 community dwelling older Mexican Americans determined that participants who changed their status from pre-frail to frail and frail to pre-frail or those who remained frail faced higher mortality risk than those who remained non-frail in 15 years [36]. Another study conducted among 1353 AIDS patients found that maintained, improved, and intermittent frailty statuses are related to increased mortality [39]. A relation between frailty change and 6-year mortality was reported [38]; however, health utilization and frailty change may be associated with mortality. The combined effects of changes in frailty status and health utilization on subsequent mortality are worthy of investigation. Therefore, the present study had two objectives: (1) to determine whether frailty status at baseline and 1 year can predict changes in healthcare utilization, such as outpatient visits, emergency care visits, and hospital admission in a sample of Taiwan older adults; and (2) to analyze the combined effects of changes in frailty status and healthcare utilization on subsequent mortality.

## Methods

### Participants

**Fig. 1:**
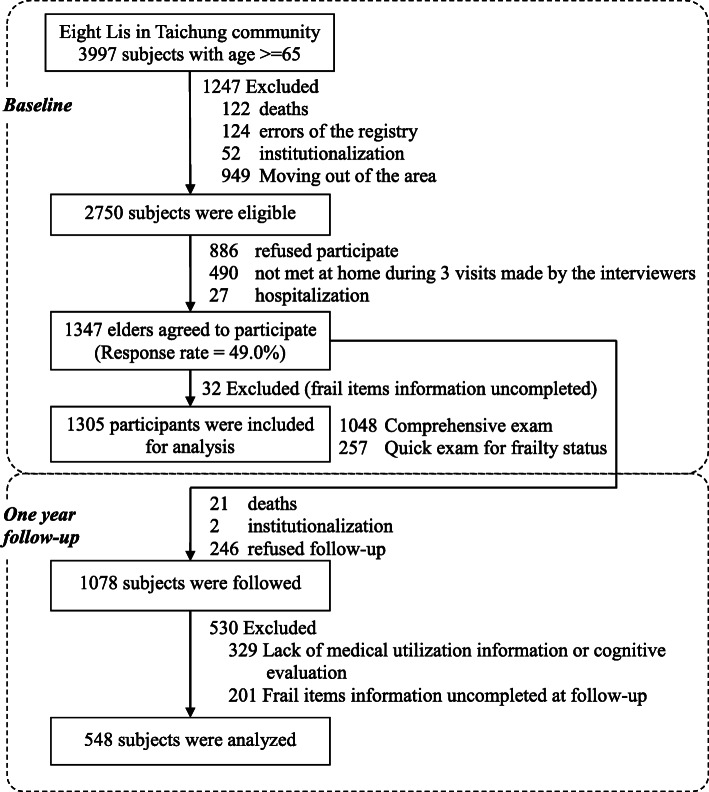
Flowchart of recruitment procedures

This work was a population-based prospective cohort study comprising 3997 residents aged ≥65 years in 8 administrative neighborhoods at the north district of Taichung City, Taiwan. It was conducted in June 2009. The age and gender distributions in the 8 administrative neighborhoods are similar to those in Taichung and Taiwan populations. All residents received recruitment letters along with the research office’s phone number. Those who called the office and agreed to participate were assigned an appointment date for the interview and physical checkup in a clinical setting*.* Individuals who were hospitalized, lived in an institution, were not at home when the interviewers visited three times, and refused to participate were excluded. Recruitment was conducted between June 2009 and August 2010. A total of 1347 individuals participated at baseline, with an overall response rate of 49.0%. In the subsequent year, 1078 older adults received follow-up. Among them, 548 subjects who provided completed frailty-related components and medical utilization information at baseline and the 1-year follow-up were included in the present study (Fig. 1).

### Frailty status and healthcare utilization measurement

Frailty status was defined on the basis of FFP [4], and it consisted of five components: shrinking, weakness, slowness, poor endurance and energy, and low physical activity level. Shrinking was characterized by an unintentional weight loss of ≥3 kg in the previous year. Weakness referred to the slowest quintile of handgrip strength in the population measured using a handgrip dynamometer (TTM-110D, TTM Co. Japan); it was based on the subgroups of gender and body mass index [40]. Slowness is measured as the slowest quintile of the population in accordance with gender and standing height subgroups and based on a 15 ft. walking time [40]. Considering the racial differences in height and body size between Western and Asian populations, we applied the cut points for weakness and slowness from a pooled analysis study of Taiwan community-dwelling older adults [41], instead of the Fried et al’s cut points, which were determined from Western populations. Endurance and energy were measured from a self-report of exhaustion and identified using two questions from the Center for Epidemiological Studies-Depression scale [42]. A low physical activity level was measured on the basis of energy expenditure in accordance with frequency, duration, and types of leisure time activity, as reported by each participant [43]. The lowest quintile of physical activity level in our study sample was identified for each gender. Participants with 0, 1–2, and ≥ 3 frailty phenotype components were considered robust, pre-frail, and frail, respectively [4]. Changes in frailty status during the 1 year period included four categories: (1) deterioration (robust at baseline and pre-frail or frail after 1 year, pre-frail at baseline and frail 1 year later); (2) unchanged pre-frail or frail; (3) unchanged robustness; and (4) improvement (frail at baseline and pre-frail or robust after 1 year; pre-frail at baseline and robust 1 year later).

Data on age, gender, marital status, education, smoking, alcohol drinking, physical behavior, and comorbidity were collected through questionnaires when the participants underwent frailty measurement. Smoking and alcohol drinking habits were categorized as never, current, and former. Regular exercise and physical activity were measured using two independent variables. Regular exercise was measured using one item through respondents’ self-report. Participants who exercised for at least 30 min three times per week during the preceding 6 months were classified as having regular exercise. Physical activity was measured frim the sum of the average time per week spent in each activity multiplied by the metabolic equivalent value.

Information regarding the outpatient visits of each specialist doctor, emergency care utilization, and hospital admission during the 2-month period before baseline and the second interview were collected through standardized questionnaires administered by an interviewer. Outpatient clinic utilization was categorized into two subcategories: rehabilitation and non-rehabilitation. This secondary data analysis study was conducted after obtaining approval from the Research Ethics Committee of China Medical University Hospital. All the methods were performed in accordance with relevant guidelines and regulations.

### Survival assessment

All-cause mortality was evaluated from the date of the second interview until August 31, 2019. Deaths were ascertained using computer linkage with a unique identification number in the national database from the Health and Welfare Data Science Center, Ministry of Health and Welfare. The records of all the deaths of Taiwan citizens are stored in this database and coded from death certificates. Survival time was defined as the time between the second interview and the date of death or the study’s end point.

### Statistical analysis

The differences in baseline characteristics among the four groups of frailty status changes within the 1-year period was identified using a chi-square test. To examine the difference of number of outpatient visits among different groups, negative binomial regression was applied because the number of outpatient visits was overdispersed with a variance-to-mean ratio of > 1. Univariate logistic regression models were used to analyze the difference of hospital admission or emergency department visit among different groups. The post hoc comparisons of medical utilization among the three groups of baseline frailty status and among the four groups of frailty status changes was tested using Bonferroni correction. Then, multivariate logistic regression models were used to determine whether frailty status changes were independently associated with outpatient visits, outpatient visits for non-rehabilitation, and hospital admission. The models were used after controlling for age, gender, education, cognitive impairment, regular exercise, and smoking and drinking habits. The associations between frailty status and the number of outpatient visits for 2 months were explored using multivariate negative binomial regression models. The combined effects of frailty status change and outpatient utilization on 9-year mortality were investigated using the Cox proportional hazard models. The proportionality of hazard assumption was confirmed by examining the product term of each independent variable with log follow-up time. For evaluating the potential drop-out bias, sensitivity analyses were performed by using inverse probability weighting (IPW) approach. The first was to derive the predicted probability of non-dropout using a multivariate logistic regression model with covariates, including age, gender, education, cognitive impairment, regular exercise, smoking and drinking status. Then the analysis was performed on non-dropout participants using a weighted model, where the weight of each individual was the inverse of the predicted probability. Statistical analysis was performed using SAS 9.4 version (SAS, Cary, NC). Statistical significance was considered at *p* of < 0.05 in all analyses.

## Results

A total of 548 older adults were included in this study. In particular, 37 (6.8%), 232 (42.3%), and 279 (50.9%) were respectively categorized as frail, pre-frail, and robust at baseline. In the subsequent year, 73 (13.3%), 356 (65.0%), and 119 (21.7%) of the older adults had deteriorated, did not exhibit any changes, and presented improved FFP components, respectively (Table 1).

**Table 1:** Frailty status at baseline and after 1-year follow-up

	At baseline		After 1-year follow-up					
	Total		Robust		Pre-frail		Frail	
	n	%	n	%	n	%	n	%
Frail	37	6.8	4	10.8	18	48.7	15	40.5
Pre-frail	232	42.3	97	41.8	118	50.9	17	7.3
Robust	279	50.9	223	79.9	56	20.1	0	0.0

Among the 548 older adults, 313 (57.1%) were male; 208 (38.0%), 140 (25.5%), and 200 (36.5%) were ≤ 70 years old, 71–75 years old, and > 75 years old, respectively; 403 (73.4%) were married; and 385 (70.3%) received education for less than 12 years. Most of older adults have regular exercise (77.8%) and didn’t smoke (78.6%) and didn’t drink (76.6%). The most common chronic diseases was hypertension (51.6%,) (Table 2).

**Table 2:** Relationship between the change in frailty status and sociodemographic factors, health related practice, and disease history

		Change of frailty status#				
	Total subjects	Deterioration	Unchanged pre-frail or frail	Unchanged robustness	Improvement	
Variable at baseline	n (%)	n (%)	n (%)	n (%)	n (%)	*χ*^*2*^ test *p-*value
Gender						**0.004**
Women	235 (42.9)	25 (34.3)	43 (32.3)	109 (48.9)	58 (48.7)	
Men	313 (57.1)	48 (65.8)	90 (67.7)	114 (51.1)	61 (51.3)	
Age						
≤ 70 years	208 (38)	25 (34.3)	31 (23.3)	110 (49.3)	42 (35.3)	
71–75 years	140 (25.5)	13 (17.8)	27 (20.3)	68 (30.5)	32 (26.9)	
> 75 years	200 (36.5)	35 (48.0)	75 (56.4)	45 (20.2)	45 (37.8)	
Marital status						0.874
Married	403 (73.5)	52 (71.2)	101 (75.9)	162 (72.7)	88 (73.9)	
Others^a^	145 (26.5)	21 (28.8)	32 (24.1)	61 (27.4)	31 (26.1)	
Education						**0.012**
Illiterate	50 (9.1)	9 (12.3)	10 (7.5)	15 (6.7)	16 (13.4)	
≤ 6 years	135 (24.6)	17 (23.3)	45 (33.8)	40 (17.9)	33 (27.7)	
7–12 years	200 (36.5)	22 (30.1)	47 (35.3)	92 (41.3)	39 (32.8)	
≥ 13 years	163 (29.7)	25 (34.3)	31 (23.3)	76 (34.1)	31 (26.1)	
Regular exercise						**< 0.001**
No	121 (22.2)	13 (18.1)	59 (44.4)	14 (6.3)	35 (29.7)	
Yes	424 (77.8)	59 (81.9)	74 (55.6)	208 (93.7)	83 (70.3)	
Smoking						**0.001**
No	430 (78.6)	59 (80.8)	89 (67.4)	189 (84.8)	93 (67.4)	
Yes	41 (7.5)	9 (12.3)	11 (8.3)	13 (5.8)	8 (8.3)	
Former	76 (13.9)	5 (6.9)	32 (24.2)	21 (9.4)	18 (24.2)	
Drinking						**0.011**
No	419 (76.6)	56 (76.7)	99 (75.0)	169 (75.8)	95 (79.8)	
Yes	86 (15.7)	14 (19.2)	14 (10.6)	43 (19.3)	15 (12.6)	
Former	42 (7.7)	3 (4.1)	19 (14.4)	11 (4.9)	9 (7.6)	
Cognitive impairment						0.100
No	520 (94.9)	70 (95.9)	124 (93.2)	217 (97.3)	109 (91.6)	
Yes	28 (5.1)	3 (4.11)	9 (6.8)	6 (2.7)	10 (8.4)	
***Disease history***						
Heart disease						0.679
No	388 (72.4)	52 (71.2)	90 (70.3)	164 (75.2)	82 (70.1)	
Yes	148 (27.6)	21 (28.8)	38 (29.7)	54 (24.8)	35 (29.9)	
Hypertension						**0.037**
No	263 (48.4)	32 (43.8)	54 (41.9)	124 (55.9)	53 (44.5)	
Yes	280 (51.6)	41 (56.2)	75 (58.1)	98 (44.1)	66 (55.5)	
Diabetes						**< 0.001**
No	442 (81.5)	58 (79.5)	94 (70.7)	196 (89.5)	94 (80.3)	
Yes	100 (18.5)	15 (20.6)	39 (29.3)	23 (10.5)	23 (19.7)	
Hyperlipidemia						0.757
No	400 (74.8)	55 (77.5)	99 (76.2)	157 (72.4)	89 (76.1)	
Yes	135 (25.2)	16 (22.5)	31 (23.8)	60 (27.7)	28 (23.9)	
Frail at baseline*						**< 0.001**
Frail	37 (6.8)	0 (0.0)	15 (11.3)	0 (0.0)	22 (18.5)	
Pre-frail	232 (42.3)	17 (23.3)	118 (88.7)	0 (0.0)	97 (81.5)	
Robust	279 (50.9)	56 (76.7)	0 (0.0)	223 (100)	0 (0.0)	

The numbers in bold indicate statistically significant *p-*values

* Fried et al. proposed the definition of frailty status with the following components: shrinking, weakness, poor endurance and energy, slowness, and low physical activity level

#Changes in frailty status during 1-year period with the following categories: improvement of frailty status, no change between baseline and follow-up, and deterioration for frailty status

^a^Others include widowed, divorced, separated, and single

The missing number for each variable is 3 in regular exercise, 1 in smoking habit, 1 in drinking habits, 12 in heart disease, 5 in hypertension, 6 in diabetes, and 13 in hyperlipidemia

Older adults with improved frailty status outnumbered those with deteriorated status. Gender, age, marital status, educational level, regular exercise, smoking and drinking habits, hypertension history, diabetes history, and frailty status at baseline were significantly associated with frailty status changes (all *p* values are < 0.05). Older adults with deteriorated FFP status were men, aged ≥75 years, educated for ≥7 years, had regular exercise, engaged in smoking and drinking, and had a history of hypertension and diabetes mellitus compared with older adults with improved FFP status (Table 2).

The baseline frailty status suggested that frail and pre-frail older adults reported significantly higher geometric mean numbers of 2-month outpatient visits and non-rehabilitation outpatient visits than robust older adults (1.1 ± 7.6 and 0.9 ± 6.4 for fail; 0.6 ± 8.4 and 0.6 ± 8.3 for pre-frail; 0.4 ± 9.3 and 0.4 ± 9.4 for robust older adults, respectively; Table 3). Changes in medical utilization between baseline and follow-up are similar among the frailty status change groups (Appendix, Table A1). Therefore, the difference in medical utilization at follow-up among these groups was explored. Older adults with unchanged pre-frail or frail status had significantly higher geometric mean numbers of 2-month outpatient and non-rehabilitation outpatient visits than those with deteriorated, improved, and unchanged robust status (0.8 ± 7.5 and 0.8 ± 7.0 for unchanged pre-frail or frail; 0.7 ± 7.1 and 0.7 ± 7.1 for deteriorated; 0.5 ± 9.6 and 0.5 ± 9.4 for improved; and 0.4 ± 9.8 and 0.3 ± 9.6 for unchanged robust older adults, respectively). There was no difference in the number of rehabilitation outpatient visits, proportions of hospital admission and emergency room visits among older adults with different baseline frailty status or change of frailty status (Table 3).

**Table 3:** Medical utilization in 2 months at 1-year follow-up among the elderly with different baseline frailty status and change of frailty status

	Total n	Number of outpatient visit			Hospitalization admission	Emergency room utilization
		Total visits	Non-rehabilitation	Rehabilitation		
		Geometric mean ± SD	Geometric mean ± SD	Geometric mean ± SD	n (%)	n (%)
Baseline frailty status*						
Frail	37	1.1 ± 7.6 ^a^	0.9 ± 6.5 ^a^	0.0 ± 8.6	1 (2.7%)	1 (2.7%)
Pre-frail	232	0.6 ± 8.4^a^	0.6 ± 8.3 ^a^	0.0 ± 2.2	5 (2.2%)	1 (0.4%)
Robust	279	0.4 ± 9.4	0.4 ± 9.3	0.0 ± 2.1	0 (0.0%)	3 (1.1%)
*p-*value^+^		**< 0.001**	**< 0.001**	0.281	0.977	0.427
Change of frailty status^#^						
Deterioration	73	0.7 ± 7.1	0.7 ± 7.1 ^c^	0.0 ± 1.0	1 (1.4%)	0 (0.0%)
Unchanged pre-frail or frail	133	0.8 ± 7.5	0.8 ± 7.0 ^c^	0.0 ± 3.6	3 (2.3%)	1 (0.8%)
Unchanged robustness	223	0.4 ± 9.8 ^b^	0.3 ± 9.6	0.0 ± 2.3	0 (0.0%)	3 (1.4%)
Improvement	119	0.5 ± 9.6 ^b^	0.5 ± 9.4	0.0 ± 2.6	2 (1.7%)	1 (0.8%)
*p-*value^+^		**< 0.001**	**< 0.001**	0.197	0.972	0.952

The numbers in bold indicate statistically significant *p-*values

* Fried et al. proposed the definition of frailty status with the following components: shrinking, weakness, poor endurance and energy, slowness, and low physical activity level

#Changes in frailty status during 1-year period with the following categories: improvement of frailty status, no change between baseline and follow-up, and deterioration for frailty status

^+^*p*-values were calculated using univariate negative binomial regression models for the number of outpatient visits, and univariate logistic regression models for the hospitalization and emergency use

^a^ Statistically significant compared with the robust group at baseline using pairwise comparisons with Bonferroni correction (*p* < 0.05/3 comparisons)

^b^ Statistically significant compared with unchanged unchanged pre-frail or frail group at 1-year follow-up using pairwise comparisons with Bonferroni correction (*p* < 0.05/6 comparisons)

^c^ Statistically significant compared with unchanged unchanged robustness group at 1-year follow-up using pairwise comparisons with Bonferroni correction (*p* < 0.05/6 comparisons)

After age, gender, education, cognitive impairment, regular exercise, smoking, and drinking habits were adjusted, multivariate logistic analysis showed that all outpatient and outpatient non-rehabilitation visits were higher among those with unchanged pre-frail or frail status (odds ratios [OR]: 1.94, 95% confidence interval [CI]: 1.02–3.71 and OR: 1.99, 95% CI: 1.04–3.79, respectively), and among those with deteriorated frailty status (OR: 2.01, 95% CI: 0.97–4.18, *p* = 0.06 borderline significant, and OR: 2.05, 95% CI: 0.99–4.26, *p* = 0.05 borderline significant, respectively); but not for risks of hospital admission and emergency room visits if unchanged robust status was used as the reference group (Table 4). The association between change of frailty status and outpatient visits disappeared after further adjustment for comorbidity (Appendix, Table A2).

**Table 4:** Change in frailty status and medical utilization via the multivariate logistic regression models

Independent variables	Risk of total outpatient visits			Risk of outpatient visits for non-rehabilitation			Risk of hospitalization admission			Risk of emergency room utilization		
	OR	95% CI		OR	95% CI		OR	95% CI		OR	95% CI	
Change of frailty status												
Deterioration	2.01	0.97	4.18	2.05	0.99	4.26	0.75	0.06	9.16	–	–	–
Unchanged pre-frail or frail	**1.94**	**1.02**	**3.71**	**1.99**	**1.04**	**3.79**	2.05	0.28	15.11	1.41	0.11	17.39
Unchanged robustness	1.00	Reference group		1.00	Reference group		–	–	–	1.00	Reference group	
Improvement	1.13	0.65	1.98	1.16	0.67	2.02	1.00	Reference group		1.13	0.10	12.64
Age (years)												
≤ 70	1.00	Reference group		1.00	Reference group		1.00	Reference group		1.00	Reference group	
71–75	**1.76**	**1.05**	**2.96**	**1.80**	**1.07**	**3.02**	2.38	0.20	28.91	2.49	0.38	16.24
> 75	**2.48**	**1.46**	**4.22**	**2.50**	**1.47**	**4.25**	1.95	0.17	22.80	–	–	–
Gender												
Women vs men	1.56	0.92	2.63	1.53	0.91	2.58	16.00	0.88	290.53	0.35	0.03	4.65
Education												
Illiterate	1.00	Reference group		1.00	Reference group		1.00	Reference group		–	–	–
≤ 6 years	1.66	0.71	3.91	1.57	0.67	3.69	0.79	0.06	11.18	1.00	Reference group-	
7–12 years	1.06	0.48	2.36	1.06	0.48	2.36	0.51	0.03	10.09	0.65	0.04	10.00
≥ 13 years	1.19	0.51	2.80	1.19	0.51	2.80	2.17	0.15	31.39	0.65	0.04	10.26
Regular exercise	1.49	0.86	2.58	1.48	0.86	2.56	3.50	0.34	36.21	–	–	–
Smoking												
No	1.00	Reference group		1.00	Reference group		1.00	Reference group		1.00	Reference group	
Yes	0.59	0.27	1.28	0.59	0.27	1.28	19.03	0.63	571.75	–	–	–
Former	1.42	0.69	2.92	1.42	0.69	2.91	4.93	0.31	78.00	4.41	0.48	40.66
Drinking												
No	1.00	Reference group		1.00	Reference group		1.00	Reference group		1.00	Reference group	
Yes	0.60	0.34	1.07	0.61	0.34	1.08	1.69	0.11	26.85	0.77	0.06	9.39
Former	0.84	0.35	2.00	0.84	0.36	2.01	–	–	–	0.88	0.06	14.18
Cognitive impairment												
Yes vs No	0.57	0.22	1.49	0.58	0.22	1.52	–	–	–	–	–	–
*Psudo-R*^*2*^		*10.7%*			*10.6%*			*26.6%*			*25.9%*	

Numbers in bold indicate statistically significant values. OR: odds ratio; 95% CI: 95% confidence interval

-: Not available due to no utilization event

**Fig. 2:**
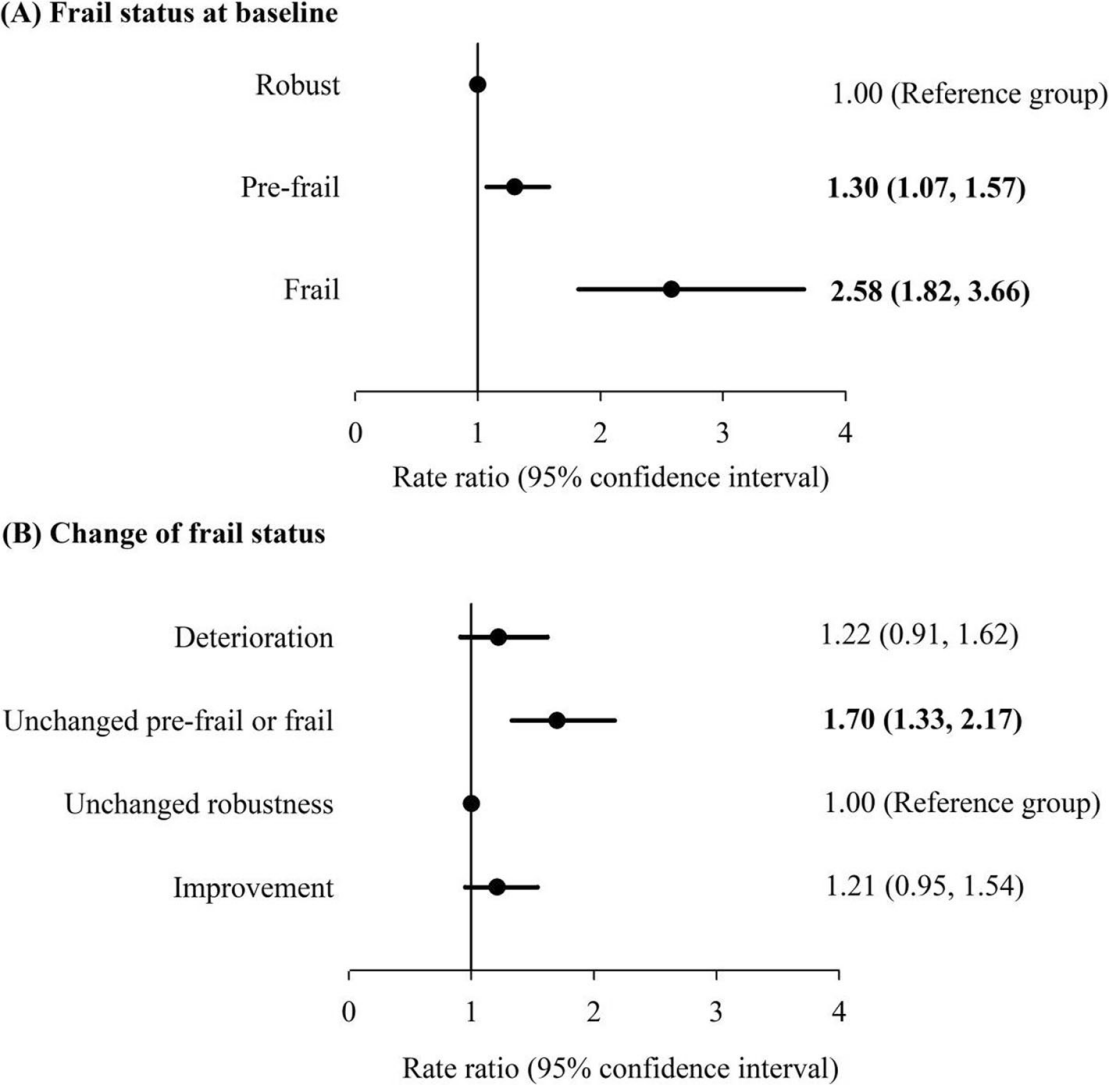
Association between frailty status and the number of outpatient visits in 2 months. Negative binomial regression models were adjusted for baseline age, gender, education, cognitive impairment, regular exercise, smoking and drinking status

Compared with those of robust older adults at baseline, the adjusted rate ratios of frail and pre-frail older adults were 2.58 [95% CI: 1.82–3.66] and 1.30 [95% CI: 1.07–1.57] in the negative binomial model, respectively. Frail older adults had approximately 141% higher number of outpatient visits in 2 months than robust older adults (*p* < 0.05; Fig. 2).

The independent effects of change in frailty status and the utilization of 2-month outpatient clinic on mortality were examined. Only the independent effects of frailty either at baseline or 1-year were found, but the independent effect of utilization outpatient clinic at 1-year follow-up was not exist (Appendix, Table A3). Furthermore, the combined effects of changes in frailty status and in the number of outpatient visits on 9-year mortality were explored using univariate and multivariate Cox proportional hazard models. The median follow-up duration was 8.83 years (at the end of August 2019). Given that the mortality of the pre-frail to robust and frail to pre-frail/ robust group was different, the groups were separated from the improvement group according to the change in frailty status (Table 5). Among the groups, the group with unchanged robustness and low outpatient clinic utilization had the lowest mortality rate (12.5%), and the frail to pre-frail/robust group and high outpatient clinic utilization group had the highest mortality rate (75.0%). Individuals with high outpatient clinic utilization had relatively higher mortality than those with low outpatient clinic utilization among most groups. Participants who improved from pre-frail to robust had lower mortality rate (17.9, 19.0%) compared with the deterioration (24.3, 27.8%) and unchanged pre-frail / frail group (35.3, 37.9%). After adjusting for age, gender, education, cognitive impairment, regular exercise, smoking, and drinking, individuals with high outpatient clinic utilization had significantly higher mortality than those with low outpatient clinic visits among unchanged pre-frail or frail (HR 2.79, 95% CI: 1.46–5.33) and frail to pre-frail/robust group (HR 9.32, 95% CI: 3.82–22.73) if the group with unchanged robustness and low outpatient clinic visits was used as the reference group. Either change in frailty status or high number of outpatient visits was related to increased mortality. The combined association of change in frailty status and outpatient utilization with mortality remained statistically significant after additional adjustment for comorbidity (hypertension and diabetes) (Appendix, Table A4). In addition to this, sensitivity analysis using IPW approach was performed for controlling potential drop-out bias. (Appendix, Table A5, Table A6, and Fig. A1). Some of these analyses using IPW approach yielded comparable results, some of them became attenuated, and some of them became significant with direction similar to the original analyses.

**Table 5:** Combined effects of change in frailty status and utilization of 2-month outpatient clinic on 9-year mortality via the Cox proportional hazard models

Change of frailty status	Utilization of outpatient clinic^a^	N	Mortality	HR (95% CI)	HRadj (95% CI)
Deterioration	Low	37	24.3%	2.03 (0.93, 4.47)	1.57 (0.70, 3.52)
	High	36	27.8%	**2.31 (1.05, 5.07)**	1.60 (0.71, 3.62)
Unchanged pre-frail or frail	Low	68	35.3%	**2.96 (1.63, 5.39)**	1.79 (0.92, 3.46)
	High	65	36.9%	**3.59 (1.99, 6.51)**	**2.79 (1.46, 5.33)**
Unchanged robustness	Low	160	12.5%	1.00 (Reference)	1.00 (Reference)
	High	63	14.3%	1.16 (0.53, 2.55)	1.33 (0.60, 2.95)
Improvement (pre-frail to robust)	Low	58	19.0%	1.56 (0.75, 3.26)	1.30 (0.61, 2.77)
	High	39	17.9%	1.50 (0.63, 3.54)	1.12 (0.46, 2.74)
Improvement (frail to pre-frail/robust)	Low	10	30.0%	2.98 (0.89, 10.04)	1.71 (0.48, 6.14)
	High	12	75.0%	**9.96 (4.52, 21.92)**	**9.32 (3.82, 22.73)**

*HR* Hazard ratio, *CI* Confidence interval. The model was adjusted for baseline age, gender, education, cognitive impairment, regular exercise, smoking and drinking habits

^a^Utilization of outpatient is categorized as “High” if the number of outpatient clinic use in 2 months is > 1 times and “Low” if the use is 1 or 0

## Discussion

This study investigated the relationship between baseline and 1-year frailty status changes and healthcare utilization. Results show that frailty status, either at baseline or 1-year change, is associated with healthcare utilization during outpatient visits, which may possibly increase medical expenditures.

In our study, frail older adults reported significantly higher proportions of outpatient and non-rehabilitation outpatient visits. Several previous studies showed that frailty is positively associated with healthcare utilization, such as outpatient clinic visits or doctor consultation [18–21] and hospital admission, and these results are consistent with the present findings [18, 20, 24, 25]. However, the present study did not reveal the association between frailty status and hospital admission or emergency room visits and is inconsistent with the previous studies [18, 23, 24]. In this study, only 6 (1.1%) and 5 (0.9%) participants were admitted to the hospital or visited the emergency department during the 2-month period before the interview. The number of participants who have hospital admission and emergency department use was very limited, which cannot meet the required number of participants for inadequate statistical power. The baseline frailty status is not associated with outpatient rehabilitation visits in our study. However, a previous study suggested that rehabilitation is effective in frail and pre-frail older adults [44]. Frail older adults should receive rehabilitation to improve their frailty status in the future.

Aside from the baseline frailty status, related changes are also associated with healthcare utilization in our study. Compared with older adults with unchanged robustness, those with unchanged pre-frail or frail were associated with increased risk of outpatient clinic visits and non-rehabilitation use. Sirven et al. used the frailty index to define frailty and concluded that elevated frailty index is associated with the increase in the number of specialist practitioners visit [35]. This finding is consistent with our results. In the present study, all participants obtained the result of their own frailty screen test. Given the convenient and cheap medical environment in Taiwan, pre-frail and frailty older adults may visit doctors to find the reversible causes of frailty and adjust their diet or increase exercise and physical activity. Such steps may result in changes in frailty status and medical utilization. If the health status of pre-frail or frail elders does not change, the use of outpatient clinic will be continuous due to health need.

In addition, the combined effect of frailty change and health utilization on 9-year all-cause mortality was observed. In this cohort, the 1-year change in frailty status and the 6-year all-cause mortality are related [38]. Other previous studies also revealed the relationship with frailty transition and mortality [36, 37, 39]. Furthermore, our findings indicated that the combined effects of the change in frailty status and outpatient utilization on 9-year mortality were significant. The present results indicate the hazard ratios of mortality were greater among elderly with high outpatient clinic utilization and with either improvement from frail to pre-frail/robustness or unchanged frailty status than those among elderly with low outpatient clinic utilization and unchanged robustness. Given the same change of frailty status, higher mortality rate was found in older adults with high healthcare utilization than in those with low utilization. A possible explanation for the phenomena is that high healthcare utilization may raise the risk of adverse outcome due to polypharmacy if elderly care is not integrated well in practice. A previous study reported the prevalence of potential drug-drug interactions in Taiwan was 25% [45]. It was implied that older adults with high health utilization are potentially at increased risk of polypharmacy and drug interactions which is more likely to experience adverse outcome. In general, high medical utilization might reflect the increasing medical needs of the elderly. For the elderly with frailty and frequent outpatient clinic visits, integrated geriatric medicine practice is needed. Doctors might need to address polypharmacy, manage sarcopenia, and find out the treatable causes of weight loss and the causes of exhaustion to improve their frailty status to decrease mortality [46].

However, we observed that individuals who had improved frail status and were frail at baseline and high outpatient clinic utilization had the highest mortality rate. In addition, individuals with improvement of frail status and low outpatient clinical utilization were associated with an increased risk of mortality in sensitivity analysis with IPW. One possible explanation is that elderly people with improved frail status may have underlying illness resulting in high risk of death. Another possible explanation is the improvement of these older adults might not sustain, because the frailty status is a dynamic stage with frequent transitions over time [47]. For clinical practice and future study, frail status in older adults should be regularly monitor. It can be considered as relative stable status if the change of frailty at two years have been observed consistently.

Participants who improved from pre-frail to robust had relatively lower mortality rate than rate of adults improved from frail to prefrail/ robust. Baseline frailty status seemed to have stronger predictive effect than frailty change. To decrease the mortality of the older adults, we should try to prevent frailty in the population by monitoring physical reserve, performing regular exercise, vaccinating for preventable diseases, undergoing prehabilitation before anticipated loss and using comprehensive geriatric evaluation and management [48].

This study has three main advantages. This work is a population-based study and the first to investigate the association between FFP status changes and medical utilization. This study is also the first to investigate the combined effect of frailty transition and medical utilization to mortality. This study has several main limitations. First, healthcare utilization information was obtained using questionnaires; therefore, recall bias is possible. However, Short ME et al. [49] suggested that self-reported healthcare utilization can be used as a proxy when medical claims and administration data, especially yearly and monthly emergency room and inpatient admissions, cannot be obtained. In this study, self-reported bias may not be severe because participants were asked to recall their recent 2-month medical utilization only to minimize the recall task for them. A longer recall period not only increases the recall task of elderly participants, but also increases the recall bias. Brusco and Watts found 35% over–reporting when older patients were asked the numbers of general practice visits in the past 6 months compared to national insurer claims data over the same period [50]. Second, our analysis was restricted to older adults who underwent comprehensive exam at baseline and after 1 year. Those who were extremely frail and sick may not be able to follow, and sample drop-out bias may be present. However, those subjects who were excluded in the analysis of this study were more likely to die (9-year mortality rate, 37.7%). We can still detect the impact of frailty changes and healthcare utilization on mortality. We used IPW approach to control the drop-out bias and the results remained similar. Third, only the 2-month healthcare utilization information prior to the second frailty status evaluation was obtained. According to a systematic review study that evaluates the relationship between frailty and hospitalization states, the follow-up period of other studies were 10 months to 5.9 years [27]. The unchanged robust group was not admitted to the hospital, and the deteriorated group did not visit the emergency room during the 2 month study period (Table 4). Given that the present study population was relatively robust, the medical hospitalization and emergency room states of the participants were limited; this factor may have affected the statistical power. Fourth, we adjusted for as many confounders as possible to minimize the effect of potential confounders, but we cannot entirely exclude the possibility of residual confounding, such as new-onset diseases and health behavior changes in the time period between frailty measurement and mortality data collection. Fifth, our findings may not be applicable elsewhere because of differences in healthcare systems, Finally, comorbidity factors were not included in the multivariate model in contrast to those in other studies [19, 20, 29], and the underlying diseases may affect healthcare utilization. However, multimorbidity is associated with frailty [51]. If they were examined using the regression model simultaneously, multicollinearity might occur and induce bias.

## Conclusions

The conditions associated with frailty status, either at baseline or 1 year, highly affect outpatient clinic visits. Thus, healthcare utilization and expenditures may increase, and improvement in or maintenance of robustness in frailty status may decrease outpatient visits. The changes in frailty status and number of outpatient visits are related to mortality. Older adults who remain robust for 1 year have a low mortality rate. Given that frailty is a dynamic process, frailty evaluation should be performed periodically to respond fast if frailty deteriorates. Further research on the relationship of frailty transition and other outcomes, such as life quality, may be considered in the future.

## Supplementary Information

**Additional file 1: Table A1.** Medical utilization in 2 months at baseline, 1 year follow-up, and difference among the elderly with different change in frailty status after 1 year follow-up. **Table A2.** Change in frailty status and medical utilization via the multivariate logistic regression models after adjusting for baseline age, gender, cognitive impairment, regular exercise, smoking, drinking status and co-morbidity (hypertension and diabetes). **Table A3.** Independent effects of change in frailty status and the utilization of 2-month outpatient clinic on 9-years mortality via Cox proportional hazard model. **Table A4.** Combined effects of change in frailty status and the utilization of 2 months outpatient clinic on 9 years mortality via Cox proportional hazard model with additional adjustment for co-morbidity. **Fig. A1.** Sensitivity analysis for association between frailty status and the number of outpatient visits in 2 months. Negative binomial regression models with inverse probability weighting approach for controlling potential drop-out bias were adjusted for baseline age, gender, education, cognitive impairment, regular exercise, smoking and drinking status. **Table A5.** Sensitivity analysis of change in frailty status and medical utilization via the multivariate logistic regression models with inverse probability weighting approach for controlling potential drop-out bias. **Table A6.** Sensitivity analysis of combined effects of change in frailty status and utilization of 2-month outpatient clinic on 9-year mortality via the Cox proportional hazard models with inverse probability weighting approach for controlling potential drop-out bias.

## Data Availability

The datasets generated and/or analyzed during the current study are not publicly available due to the policy declared by Ministry of Health and Welfare in Taiwan but are available from the corresponding author on reasonable request.

## Abbreviations

OR: Odds ratios
CI: Confidence interval
HR: Hazard ratios
FFP: Fried’s frailty phenotype
IPW: Inverse probability weighting
