# Supplementary material for: Frailty status changes are associated with healthcare utilization and subsequent mortality in the elderly population
Source: BMC Public Health. 2021 Apr 1;21:645. doi: 10.1186/s12889-021-10688-x (PMC8017879; doi:10.1186/s12889-021-10688-x)
Supplement: Supplementary file 1 — Additional file 1: Table A1. Medical utilization in 2 months at baseline, 1 year follow-up, and difference among the elderly with different change in frailty status after 1 year follow-up. Table A2. Change in frailty status and medical utilization via the multivariate logistic regression models after adjusting for baseline age, gender, cognitive impairment, regular exercise, smoking, drinking status and co-morbidity (hypertension and diabetes). Table A3. Independent effects of change in frailty status and the utilization of 2-month outpatient clinic on 9-years mortality via Cox proportional hazard model. Table A4. Combined effects of change in frailty status and the utilization of 2 months outpatient clinic on 9 years mortality via Cox proportional hazard model with additional adjustment for co-morbidity. Fig. A1. Sensitivity analysis for association between frailty status and the number of outpatient visits in 2 months. Negative binomial regression models with inverse probability weighting approach for controlling potential drop-out bias were adjusted for baseline age, gender, education, cognitive impairment, regular exercise, smoking and drinking status. Table A5. Sensitivity analysis of change in frailty status and medical utilization via the multivariate logistic regression models with inverse probability weighting approach for controlling potential drop-out bias. Table A6. Sensitivity analysis of combined effects of change in frailty status and utilization of 2-month outpatient clinic on 9-year mortality via the Cox proportional hazard models with inverse probability weighting approach for controlling potential drop-out bias. [file 12889_2021_10688_MOESM1_ESM.docx]

**APPENDIX**

# **Table A1.** Medical utilization in 2 months at baseline, 1 year follow-up, and difference among the elderly with different change in frailty status after 1 year follow-up

|  |  | |  | |  | |  | | Improvement | |  |
| --- | --- | --- | --- | --- | --- | --- | --- | --- | --- | --- | --- |
|  | Deterioration | | Unchanged  pre-frail or frail | | Unchanged robustness | | A: Pre-frail to  robust | | B: Fail to  pre-frail/robust | | Comparison between group A & B |
|  | n=73 | | n=134 | | n=223 | | n=97 | | n=23 | |  |
| Medical utilization | Means | SD | Means | SD | Means | SD | Means | SD | Means | SD | *p*-value |
| **At baseline** |  |  |  |  |  |  |  |  |  |  |  |
| Total visits | 3.12 | 7.52 | 3.19 | 6.83 | 2.02 | 3.60 | 3.02 | 6.98 | 3.13 | 2.70 | > 0.05 |
| Rehabilitation | 1.00 | 7.14 | 1.23 | 6.30 | 0.17 | 1.72 | 1.12 | 6.58 | 0.61 | 2.02 | > 0.05 |
| Nonrehabilitation | 2.12 | 2.27 | 1.96 | 1.98 | 1.86 | 3.19 | 1.90 | 2.26 | 2.52 | 1.41 | > 0.05 |
| Hospitalization admission | 0.01 | 0.12 | 0.03 | 0.17 | 0.00 | 0.00 | 0.01 | 0.1 | 0.00 | 0.00 | > 0.05 |
| **After 1 year** |  |  |  |  |  |  |  |  |  |  |  |
| Total visits | 1.59 | 1.12 | 2.15 | 3.38 | 1.27 | 2.16 | 1.39 | 1.40 | 2.17 | 1.97 | **< 0.05** |
| Rehabilitation | 0.00 | 0.00 | 0.46 | 2.96 | 0.17 | 1.91 | 0.05 | 0.51 | 0.39 | 1.37 | > 0.05 |
| Nonrehabilitation | 1.59 | 1.12 | 1.69 | 1.28 | 1.1 | 1.1 | 1.34 | 1.32 | 1.78 | 1.24 | > 0.05 |
| Hospitalization admission | 0.04 | 0.35 | 0.02 | 0.15 | 0.00 | 0.00 | 0.01 | 0.10 | 0.04 | 0.21 | > 0.05 |
| **Difference (After**–**Baseline)** |  |  |  |  |  |  |  |  |  |  |  |
| Total visits | -1.53 | 7.35 | -1.04 | 5.31 | -0.75 | 3.81 | -1.63 | 7.11 | -0.96 | 2.20 | > 0.05 |
| Rehabilitation | -1.00 | 7.14 | -0.78 | 4.55 | 0.00 | 2.32 | -1.07 | 6.60 | -0.22 | 1.62 | > 0.05 |
| Nonrehabilitation | -0.53 | 2.48 | -0.26 | 2.24 | -0.75 | 3.13 | -0.56 | 2.55 | -0.74 | 1.39 | > 0.05 |
| Hospitalization admission | 0.03 | 0.37 | -0.01 | 0.23 | 0.00 | 0.00 | 0.00 | 0.14 | 0.04 | 0.21 | > 0.05 |

SD, standard deviation

# **Table A2.** Change in frailty status and medical utilization via the multivariate logistic regression models after adjusting for baseline age, gender, cognitive impairment, regular exercise, smoking, drinking status and co-morbidity (hypertension and diabetes)

| Independent variables | Risk of total outpatient visits | | | Risk of outpatient visits for non-rehabilitation | | | Risk of hospitalization admission | | | Risk of emergency room utilization | | |
| --- | --- | --- | --- | --- | --- | --- | --- | --- | --- | --- | --- | --- |
|  | ORadj | 95% CI | | ORadj | 95% CI | | ORadj | 95% CI | | OR | 95% CI | |
| Change of frailty status |  |  |  |  |  |  |  |  |  |  |  |  |
| Deterioration | 1.89 | 0.89 | 4.02 | 1.93 | 0.91 | 4.10 | 0.70 | 0.06 | 8.76 | - | - | - |
| Unchanged pre-frail or frail | 1.59 | 0.82 | 3.11 | 1.63 | 0.84 | 3.17 | 2.11 | 0.28 | 16.10 | 2.27 | 0.16 | 33.15 |
| Unchanged robustness | 1.00 | Reference group | | 1.00 | Reference group | | - | - | - | 1.00 | Reference group | |
| Improvement | 1.01 | 0.57 | 1.80 | 1.04 | 0.59 | 1.85 | 1.00 | Reference group | | 1.65 | 0.13 | 20.99 |

# **Table A3.** Independent effects of change in frailty status and the utilization of 2-month outpatient clinic on 9-years mortality via Cox proportional hazard model

| Independent variables | HRadj (95% CI) |
| --- | --- |
| **Model I.** |  |
| Frailty status at baseline |  |
| Robust | 1.00 (Reference) |
| Pre-Frail | **1.85 (1.19, 2.88)** |
| Frail | **4.60 (2.36, 8.97)** |
| Utilization of outpatient clinic at baseline | 1.08 (0.75, 1.56) |
| **Model II.** |  |
| Frailty status at 1-year follow-up |  |
| Robust | 1.00 (Reference) |
| Pre-Frail | **1.54 (1.02, 2.34)** |
| Frail | **3.66 (1.99, 6.72)** |
| Utilization of outpatient clinic at 1-year follow-up | 1.29 (0.88, 1.89) |
| **Model IIII.** |  |
| Frailty status changes |  |
| Deterioration | 1.30 (0.70, 2.42) |
| Unchanged pre-frail or frail | **1.82 (1.07, 3.08)** |
| Unchanged robustness | 1.00 (Reference) |
| Improvement (pre-frail to robust) | 1.06 (0.57, 1.97) |
| Improvement (frail to pre-frail/robust) | **3.44 (1.58, 7.50)** |
| Utilization of outpatient clinic at 1-year follow-up | 1.38 (0.95, 2.03) |

HR, hazard ratio; CI, confidence interval. Each model was adjusted for baseline age, gender, education, cognitive impairment, regular exercise, smoking, drinking status and co-morbidity (hypertension and diabetes)

*Utilization of outpatient is categorized as “High” if the number of outpatient clinic use in 2 months is >1 times and “Low” if the use is 1 or 0.

# **Table A4.** Combined effects of change in frailty status and the utilization of 2 months outpatient clinic on 9 years mortality via Cox proportional hazard model with additional adjustment for co-morbidity

| Change of frailty status | Utilization of outpatient clinic* | HRadj (95% CI) |
| --- | --- | --- |
| Deterioration | Low | 1.54 (0.69, 3.46) |
|  | High | 1.45 (0.63, 3.32) |
| Unchanged pre-frail or frail | Low | 1.60 (0.81, 3.14) |
|  | High | **2.51 (1.30, 4.85)** |
| Unchanged robustness | Low | 1.00 (Reference) |
|  | High | 1.26 (0.57, 2.81) |
| Improvement (pre-frail to robust) | Low | 1.28 (0.60, 2.76) |
|  | High | 1.02 (0.41, 2.52) |
| Improvement (frail to pre-frail/robust) | Low | 1.38 (0.37, 5.08) |
|  | High | **7.89 (3.14, 19.84)** |

HR, hazard ratio; CI, confidence interval. The model was adjusted for baseline age, gender, education, cognitive impairment, regular exercise, smoking, drinking status and co-morbidity (hypertension and diabetes)

*Utilization of outpatient is categorized as “High” if the number of outpatient clinic use in 2 months is >1 times and “Low” if the use is 1 or 0.

#
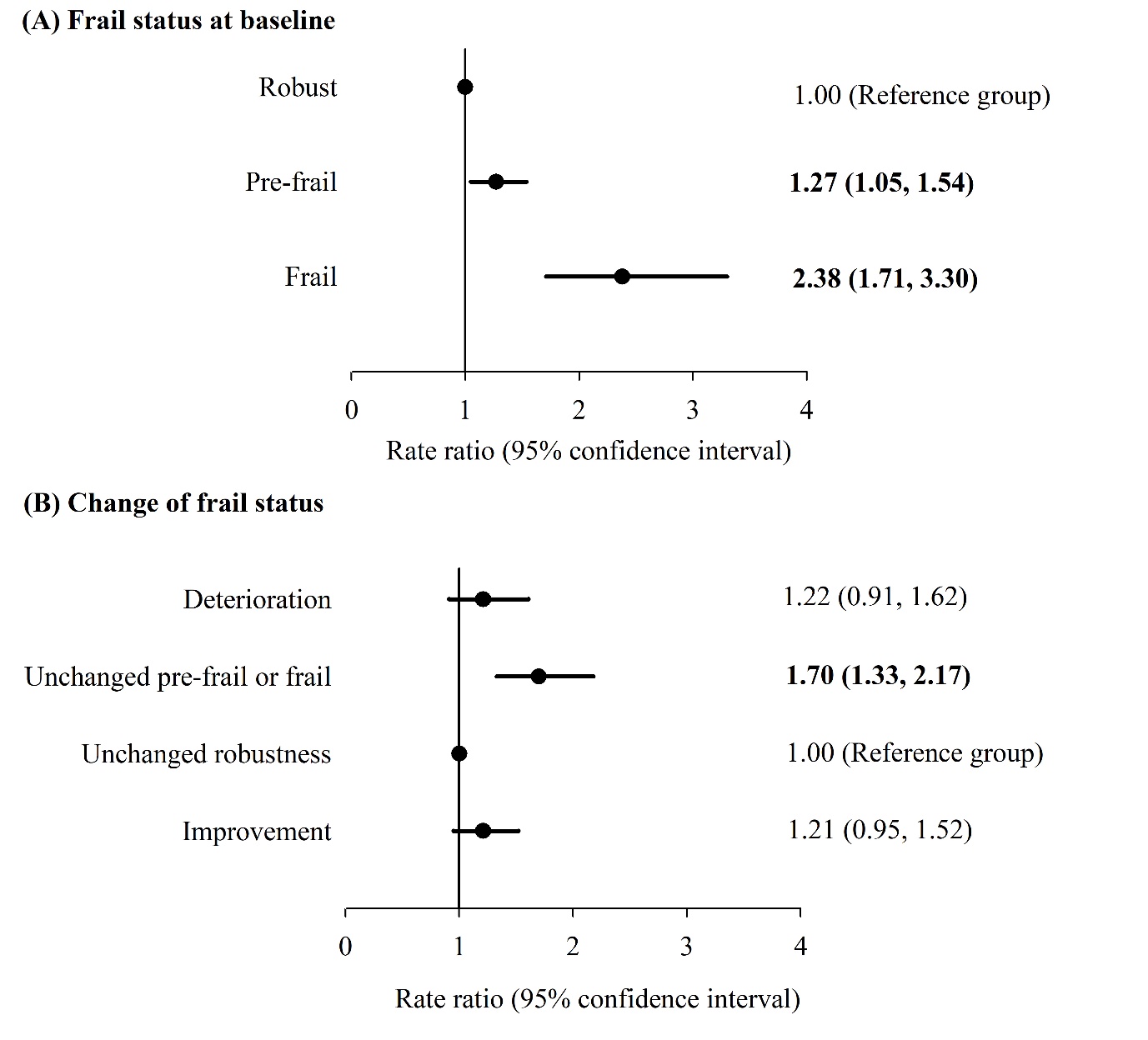


# **Fig A1.** Sensitivity analysis for association between frailty status and the number of outpatient visits in 2 months. Negative binomial regression models with inverse probability weighting approach for controlling potential drop-out bias were adjusted for baseline age, gender, education, cognitive impairment, regular exercise, smoking and drinking status

# **Table A5.** Sensitivity analysis of change in frailty status and medical utilization via the multivariate logistic regression models with inverse probability weighting approach for controlling potential drop-out bias

| Independent variables | Risk of total outpatient visits | | | Risk of outpatient visits for non-rehabilitation | | | Risk of hospitalization admission | | | Risk of emergency room utilization | | | |
| --- | --- | --- | --- | --- | --- | --- | --- | --- | --- | --- | --- | --- | --- |
|  | OR | 95% CI | | OR | 95% CI | | OR | 95% CI | | OR | 95% CI | | |
| Change of frailty status |  |  |  |  |  |  |  |  |  |  |  | |  |
| Deterioration | 1.48 | 0.72 | 3.06 | 1.52 | 0.73 | 3.13 | 0.95 | 0.10 | 9.38 | - | - | | - |
| Unchanged pre-frail or frail | 1.12 | 0.61 | 2.08 | 1.15 | 0.62 | 2.13 | 2.31 | 0.38 | 13.92 | 1.51 | 0.12 | | 18.80 |
| Unchanged robustness | 1.00 | Reference group | | 1.00 | Reference group | | - | - | - | 1.00 | Reference group | | |
| Improvement | 0.76 | 0.47 | 1.21 | 0.78 | 0.49 | 1.24 | 1.00 | Reference group | | 0.94 | 0.10 | | 8.85 |
| Age (years) |  |  |  |  |  |  |  |  |  |  |  | |  |
| ≤70 | 1.00 | Reference group | | 1.00 | Reference group | | 1.00 | Reference group | | 1.00 | Reference group | | |
| 71–75 | 0.94 | 0.62 | 1.44 | 0.96 | 0.63 | 1.46 | 3.68 | 0.36 | 37.91 | 2.77 | 0.44 | | 17.64 |
| >75 | **3.91** | **2.47** | **6.19** | **3.97** | **2.51** | **6.28** | 2.23 | 0.20 | 24.85 | - | - | | - |
| Gender |  |  |  |  |  |  |  |  |  |  |  | |  |
| Women vs men | 1.39 | 0.90 | 2.14 | 1.37 | 0.89 | 2.11 | 9.01 | 0.73 | 111.36 | 0.34 | 0.07 | | 1.65 |
| Education |  |  |  |  |  |  |  |  |  |  |  | |  |
| [Illiterate](http://tw.dictionary.yahoo.com/search?ei=UTF-8&p=%E6%96%87%E7%9B%B2) | 1.00 | Reference group | | 1.00 | Reference group | | 1.00 | Reference group | | - | - | - | |
| ≤6 years | **2.76** | **1.33** | **5.72** | **2.65** | **1.28** | **5.48** | 1.08 | 0.09 | 12.90 | 1.00 | Reference group- | | |
| 7-12 years | 0.91 | 0.46 | 1.80 | 0.91 | 0.46 | 1.80 | 0.52 | 0.03 | 9.49 | 0.72 | 0.05 | | 10.72 |
| ≥ 13 years | 1.53 | 0.72 | 3.25 | 1.52 | 0.72 | 3.24 | 2.36 | 0.18 | 30.39 | 0.68 | 0.04 | | 10.61 |
| Regular exercise | **0.61** | **0.39** | **0.95** | **0.61** | **0.39** | **0.95** | 3.79 | 0.41 | 35.10 | - | - | | - |
| Smoking |  |  |  |  |  |  |  |  |  |  |  | |  |
| No | 1.00 | Reference group | | 1.00 | Reference group | | 1.00 | Reference group | | 1.00 | Reference group | | |
| Yes | **0.38** | **0.19** | **0.77** | **0.38** | **0.19** | **0.77** | 11.80 | 0.54 | 256.21 | - | - | | - |
| Former | 1.65 | 0.83 | 3.28 | 1.64 | 0.83 | 3.26 | 3.41 | 0.26 | 44.05 | 5.65 | 0.64 | | 49.72 |
| Drinking |  |  |  |  |  |  |  |  |  |  |  | |  |
| No | 1.00 | Reference group | | 1.00 | Reference group | | 1.00 | Reference group | | 1.00 | Reference group | | |
| Yes | **0.57** | **0.33** | **0.98** | **0.58** | **0.34** | **0.99** | 1.07 | 0.08 | 13.77 | 0.62 | 0.05 | | 7.18 |
| Former | 0.61 | 0.28 | 1.33 | 0.61 | 0.28 | 1.34 | - | - | - | 0.60 | 0.04 | | 9.43 |
| Cognitive impairment |  |  |  |  |  |  |  |  |  |  |  | |  |
| Yes vs No | **0.43** | **0.26** | **0.72** | **0.44** | **0.27** | **0.73** | - | - | - | - | - | | - |
| *Psudo-R^2^* |  | *25.9%* |  |  | *25.6%* |  |  | *28.4%* |  |  | *31.7%* | |  |

Numbers in bold indicate statistically significant values. OR: odds ratio; 95% CI: 95% confidence interval

-: Not available due to no utilization event

# **Table A6.** Sensitivity analysis of combined effects of change in frailty status and utilization of 2-month outpatient clinic on 9-year mortality via the Cox proportional hazard models with inverse probability weighting approach for controlling potential drop-out bias

| Change of frailty status | Utilization of outpatient clinic* | HR (95% CI) | HRadj (95% CI) |
| --- | --- | --- | --- |
| Deterioration | Low | 2.05 (0.94, 4.44) | 1.72 (0.79, 3.77) |
|  | High | **2.40 (1.11, 5.17)** | 1.80 (0.81, 4.01) |
| Unchanged pre-frail or frail | Low | **3.07 (1.71, 5.52)** | **2.63 (1.40, 4.94)** |
|  | High | **3.67 (2.03, 6.61)** | **3.85 (2.05, 7.21)** |
| Unchanged robustness | Low | 1.00 (Reference) | 1.00 (Reference) |
|  | High | 1.16 (0.53, 2.55) | 1.38 (0.62, 3.05) |
| Improvement (pre-frail to robust) | Low | 1.56 (0.89, 2.74) | 1.45 (0.77, 2.71) |
|  | High | 0.57 (0.27, 1.2) | 0.57 (0.25, 1.32) |
| Improvement (frail to pre-frail/robust) | Low | **4.02 (2.26, 7.16)** | **4.50 (2.15, 9.42)** |
|  | High | **9.38 (4.78, 18.42)** | **14.59 (6.78, 31.36)** |

HR, hazard ratio; CI, confidence interval. The model was adjusted for baseline age, gender, education, cognitive impairment, regular exercise, smoking and drinking habits.

*Utilization of outpatient is categorized as “High” if the number of outpatient clinic use in 2 months is >1 times and “Low” if the use is 1 or 0.
